# Medical and Philosophical Intelligence

**Published:** 1811-06

**Authors:** 


### MEDICAL AND PHILOSOPHICAL INTELLIGENCE.

*Extract from the Minutes of the Jacksonian Committee, on the* 19*th of February*, 1811.—Read two other Dissertations on the same important subject (Rabies Canina); one having for its motto,

> "On dogs the infection first began,
> And last the vengeful arrows fix'd on man." *Pope's Iliad, Book* 1.

> "Beware of yonder Dog,
> Look, when he fawns he bites; and when he bites,
> His venom tooth will rankle to the death." *Shakes. Rich.* III.

the other having the motto, "Spero."

Whereupon the Committee Resolved,

That, in their judgment, such two Dissertations are highly meritorious productions, are additionally deserving consideration from the assurance, in each, that the author will continue to prosecute inquiries on the subject of Rabid Animals; and that such Dissertations are equally worthy of the Jacksonian Prize.

That it be, therefore, recommended to the Court of Assistants, on this gratifying occasion, to resolve on granting an extraordinary premium of 10*l.* in order that the author of each such Dissertations may receive a token of equal approbation of his valuable labour.

That as, however, the Jacksonian Prize must be awarded to one of the Candidates, and as it is a special duty of the Committee to make such award, that such prize be adjudged to the author of the Dissertation with the motto "On Dogs, &c."

That, if the proposition for an extraordinary premium should be agreed to by the Court, it is the opinion of the Committee that such premium should be given to the author of the Dissertation with the motto "Spero."

*Extract from the Minutes of the Court of Assistants, on the 11th of April*, 1811.—It was then moved, seconded, and resolved, that the Jacksonian Prize for the year 1810, adjudged to Mr. John Smith Soden of Coventry, and Mr. James Gillman of Highgate, be delivered to them by the Board of Curators; at such time and in such manner as that Board shall judge proper.

---

Urinary Worms.—M. Pepion, Physician to the Navy at Cherbourg, has lately related the particulars of a case in which the patient voided a number of small worms in his urine. He describes them to be three lines in length, of extreme tenuity, the head round and red; the tail red and bifurcated; two antennæ; four feet; the body consisted of yellow rings. The most effectual remedy for them, was a paste formed of the seeds of citron, in doses of half a drachm, three or four times a day.—*Bulletin des Sciences Medicales.*

---

Use of Savine in Gout.—M. Hufeland, for several years, has employed Savine with great success in all forms of Chronic Gout, whether in the head, the chest, or the extremities, even when the complaint has resisted the most energetic remedies, as guaiacum, sulphur, antimony, and mercury. Savine does not impair the digestive functions; but great caution is requisite in administering it, on account of its acting, like cantharides, on the urinary organs, and on the uterus, whence may result pain and inflammation in the kidneys and in the bladder, strangury, hæmatury, and uterine hæmorrhage. It has proved efficacious in the form of powder, in doses of ten grains to a scruple, in 24 hours. It has also succeeded in decoction; and the essential oil may be given with safety, but on account of its great strength, the dose should not exceed a single drop triturated with sugar, and taken at two separate times, morning and evening. When five or six drops have been taken in the day, inflammatory symptoms of the kidneys and bladder have proved very distressing.

By

By an imperial decree, dated Palace of Bois-le-duc, May 7th, 1810, Napoleon has offered a prize of a *million* of francs for the best machine for spinning flax. The money is deposited in the hands of the minister of the interior, and will be awarded *à l'inventeur de quelque nation qu'il puisse être, de la meilleur machine propre à filer le lin.*

*Palace of St. Cloud, June* 18*th*, 1810.

Sr. Proust has received 100,000 fr. and Sr. Fouques a sum of 40,000 fr. from the Emperor, as a gratification and encouragement for their discovery of the process of making sugar from raisins.

They are enjoined to employ this money in establishing manufactories of sugar from raisins, in such parts of the southern department as the minister of the interior shall appoint.

They are required to explain their process, which is to be published and forwarded to all the vinegar departments.

From the 1st of January, 1811, the sugar of raisins is to be used instead of that from the sugar-cane in all public establishments.

Napoleon has offered 100,000 fr. for the discovery of an indigenous plant of easy culture, capable of being substituted for Indigo, in price, use, permanency, and brilliancy of colour.

An equal sum will be awarded to whoever shall furnish a process for fixing an indigenous vegetable colour, on cotton, wool, linen, silk, &c. so as to replace indigo.

50,000 francs will be given to him who shall discover the means of mixing indigo with indigenous substances, or of employing it in a new manner, so that half the quantity will produce the full effects in intensity and solidity of colour.

25,000 francs will be given for ascertaining the means of diminishing the quantity of indigo one fourth.

25,000 francs will be given for the discovery of a certain and easy process for extracting, from the plant which furnishes woad (satis tinctoria Linn.) the colouring matter, and employing it as a dye.

100,00 fr. will be further given if this colouring matter can be made to impart the fineness and brilliancy of indigo, without its durability being injured.

25,000 fr. will be given for the discovery of a certain and easy mode of dying wool and silk with Prussian blue, so as to obtain an uniform and brilliant colour, unchangeable by rubbing or washing. The competitors for this prize are to send a description of their process with some specimens of dyed stuffs, &c. to the minister of the interior.

---

Mr. Davy has lately read an account (at the Royal Institution) of a meteoric stone, which fell a few weeks since in the county of Tipperary. The phenomena attending its fall were the same as described in other instances of the like kind. The stone has been analyzed by Mr. Higgins of Dublin, and contains, like other meteoric stones, iron and nickel. This ingenious philosopher and chemist, when endeavouring to explain the phenomenon of meteoric stones, observed, that hydrogen gas, or inflammable air, will dissolve some of the metals, and form, with them, an invisible metallic gas. When this gas explodes, the metals are deposited, in a metallic form, on the sides of the vessel which contained the gas. He exhibited arsenic, tellurium, and sodium, sodium, in a gaseous state combined with hydrogen. When the hydrogen was inflamed, by a mixture of oxymuriatic gas, the metals were deposited in a solid form, coating the sides of the vessels. As the stones which had fallen from the atmosphere were all metallic, he inferred that they might be formed by an explosion of a large quantity of inflammable air, in which the metals had been dissolved. The opinion that these meteoric stones are formed from metals in a gaseous state, which explode and condense, seems very probable, and is agreeable to the appearances which accompany their fall. The dense cloud, the loud explosion, the brilliant light which precedes their descent, the heat of the stones when they are first found, are all indications of their being thus produced. Mr. Davy was, however, inclined to trace their origin to another source, and to consider them as the ruins of some planetary body; or, perhaps, they might be small satellites moving round some of the planets, which, coming near the orbit of the earth, were attracted to it. Though this opinion has been advanced before by some philosophers on the continent, it is too improbable, and wildly conjectural, to be at all admitted.

---

An article in a late Martinico gazette describes the effects of a tree growing on the coasts of that island, under the name of the divine *Alconorque.* It is said to be a specific in diseases of the viscera, especially of the liver and lungs. An infusion of the outer bark is the way in which it is used. A glass of this infusion is taken morning and evening with two spoonfuls of honey. Milk, acids, spices, and all irritating substances are to be avoided during its use. A cataplasm of it is used for pains in the region of the liver. This account of the virtues of the divine *Alconorque,* is said to be derived from the Indians. What is the divine *Alconorque?* and who are the Indians of Martinique?

---

Dr. Terry, of Coventry, has recently given the Oleum Terebinthinæ in a case of tænia with success. The patient, a lady above fifty years of age, being in a weak state of health, Dr. T. did not give the remedy in the large doses, which some practitioners have employed. He directed a tea-spoonful to be taken every four hours, mixed with honey. The third dose acted gently on the bowels, and brought away some detached portions of tænia which measured about four yards in length. Before she had consumed an ounce of the oil, a worm was discharged, rolled into a ball. She did not unfold it to ascertain its length; having seen what she supposed to be the head. The oil did not produce any uneasy sensation in the stomach, nor any irritation in the urinary passages.
In another case of tænia Dr. Terry prescribed the oleum terebinthinæ in a similar manner, and with like success; a worm being expelled that measured more than nine yards.

---

*Inflammatory Diseases.*—General and Topical Bleeding. Query. Would it not be of use to follow it up immediately with strong stimulants? It appears so in the external inflammation, as to the eye. Query, in internal inflammation? *Dr. Beddoes' Adversaria.*
When Dr. Beddoes made this query, he was not aware that in Pneumonic inflammation in the thorax, some veterinary surgeons pursue precisely cisely the practice here suggested. Immediately after one copious bleeding, the cuprum vitriolatum is given in large and repeated doses. This practice, apparently so contrary to all principle, is said to be completely successful, very few horses dying when thus treated; while the evacuating treatment is unfortunate in a much greater proportion. The rationale of this, if it can be said to have any rationale, is, that after the vessels are excessively depleted, for the single abstraction of blood is enormous, a degree of tone or constriction is given to them by the cuprum vitriolatum, which prevents congestion of blood again occurring in the lungs, or a serous effusion taking place. Whatever may be the theory of this practice, the fact itself deserves notice.

---

Dr. Adams commences his Summer Course of Lectures on the Institutes and Practice of Medicine, the beginning of June, at his house No. 17, Hatton Garden.

---

Mr. A. T. Thompson has commenced his Summer Course of Lectures on the Science of Botany. A lecture is delivered every morning, except on Saturdays and Sundays, at nine o'clock, in the library of the Botanic Garden, Sloan Street; and the course is particularly designed for Medical students.

---

Mr. W. T. Brande's Lectures on the principles of Experimental Chemistry, commenced on the 28th of May, at the Theatre of Anatomy, Great Windmill Street.

---

Dr. Tuthill will begin his Summer Course of Lectures, on the Practice of Physic, and on the Laws and Operations of Chemistry, with particular examinations to facilitate the acquisition of Medical and Chemical knowledge, on Monday the 3d June, at his house in Soho Square.

---

Dr. Merriman, Physician, Accoucheur to the Middlesex Hospital, and Westminster General Dispensary, proposes, during the Summer, to deliver a Course of Lectures on the Theory and Practice of Midwifery, and on Female and Infantile Diseases.

---

Dr. Hooper and Dr. Agar recommence their Lectures on the Theory and Practice of Physic, Chemistry, and the Materia Medica, at the Theatre in Cork Street, on Monday, June 3, at eight o'clock in the morning.

---

Dr. Haighton's first Course of Lectures on Midwifery, comprehending the Diseases of Women and Children, commences this day, June 1, at the Theatre, Guy's Hospital.

---

Mr Brooks' Summer Course of Lectures on Anatomy, will commence, at the Theatre of Anatomy, Blenheim Street, on the 8th of June.

