# Corrigenda

**Published:** 1811-06

**Authors:** 


					543, 1. 3S, before Extract from, insert K.OYAL Uolleg*
or Surgeons, London.
Printed by E, limited, Great New Street, Fetter JLant.

				

